# Individual-centric N-of-1 trials: a case study assessing the effect of alcohol abstinence on mood levels

**DOI:** 10.1186/s12874-025-02738-4

**Published:** 2026-01-07

**Authors:** Marco Piccininni, Jascha Wiehn, Stefan Konigorski

**Affiliations:** 1 Digital Health - Machine Learning Research Group, Hasso Plattner Institute for Digital Engineering, Prof.-Dr.-Helmert-Straße 2 -3, 14482 Potsdam, Germany; 2 Digital Engineering Faculty, University of Potsdam, Potsdam, Germany; 3 Charité – Universitätsmedizin Berlin, Berlin, Germany

**Keywords:** N-of-1 trial, Individual-centric, Alcohol, Mood, Causal inference

## Abstract

**Background:**

Popularized in the 1980s, N-of-1 trials have emerged as a useful study design to assess the effects of interventions in single individuals. This study design consists of observing outcomes over time for the same individual under periods of exposure to an intervention and a comparator. Despite the simple idea, N-of-1 trials can require strong assumptions in the analysis phase to identify and estimate causal effects. As an illustrative example, we present an N-of-1 trial aiming at assessing the effect of alcohol abstinence on mood.

**Methods:**

The N-of-1 trial participant decided to join a month-long nationwide alcohol abstinence campaign and was interested in the effects of alcohol abstinence on his mood. Every eight hours, the participant collected data about his own mood levels, number of alcohol units consumed, and social interactions, before, during, and after the alcohol abstinence period. Mood levels were measured using a 5-point Likert scale ranging from -2 to 2. To analyze the N-of-1 trial data, we relied on an explicit causal framework and made precise assumptions about the data generating process. We used a g-computation algorithm to estimate, for each time point, the individual-specific difference between the expected mood outcomes under the “always abstain from alcohol” intervention and “always drinking as usual” comparator.

**Results:**

Overall, 264 time points were recorded, 171 under no intervention, and 93 during the intervention (alcohol abstinence) period. After adjusting for the other time-varying causes of mood, no statistically significant effect of alcohol units on mood level was found for measurements at the same time point; however, the number of alcohol units reported had a statistically significant negative effect on mood levels at the subsequent time point. The mean of the individual-specific average treatment effects across the entire study period was 0.05 (95%CI: -0.06, 0.15).

**Conclusions:**

N-of-1 trials can be truly individual-centric studies, tailored to the needs and preferences of the participants. Analyzing data from N-of-1 trials can be complex, and the use of a causal framework can help inform the analyses.

## Background

Randomized control trials (RCTs) are the gold standard for quantifying the effect of a treatment on an outcome [1–7]. Randomization provides exchangeability between treatment groups, allowing for the identification of causal effects [1]. When conducting an RCT, the typical aim is to estimate the so-called “average treatment effect”, which is a contrast of the average of the outcomes if all individuals in the population had been treated versus the average of the outcomes if no individual had been treated [1, 6, 8]. However, the fact that a treatment is effective on average in a population does not necessarily imply that it is effective for all individuals in that population [3–6, 9, 10]. The attempt to personalize treatment decisions based on individuals’ characteristics has motivated great interest in precision medicine during recent decades [3].

N-of-1 trials have been described as the “ultimate strategy for individualizing medicine” [11]. Popularized in the 1980 s [12], N-of-1 trials are within-person crossover trials that allow the estimation of individual-specific effects [6, 7]. The term N-of-1 trial is sometimes used to refer specifically to randomized and double-blinded trials [7]. In this work, we use a broader definition, using “N-of-1 trial” to denote any one-person study in which the participant sequentially undergoes treatment and non-treatment periods with at least one crossover, according to a pre-specified protocol, while outcome measurements are regularly collected.

Despite the limited applicability in terms of research questions [7], N-of-1 trials readily cater to personalization and tailoring to the individual; in contrast to an RCT, there is no need to forcefully regularize treatment schedules or outcome definitions across multiple individuals [5].

The N-of-1 trial design can be truly individual-centric: individuals or patients can set up their own individual trials to answer causal questions that are relevant to them, taking into account their schedules, their treatment preferences, and considering outcomes that are of personal importance. In recent years, with the emergence of new technologies and software, it has become much easier for patients to design their own N-of-1 trials and collect data [6, 13]. However, despite the simple study design, meaningfully analyzing N-of-1 trial data can be complex and may require commitment to specific statistical frameworks and strong assumptions about the data generating process to obtain valid effect estimates [4, 14, 15].

The aim of this study is to demonstrate the suitability of N-of-1 trials to answer individual-specific causal questions and to highlight the importance of making assumptions explicit to obtain causally interpretable results. To achieve this aim, we present a case study of an individual-centric N-of-1 trial aiming at assessing the effect of alcohol abstinence on mood. We describe the participant’s motivation for conducting the trial, outline the study design, detail the chosen statistical framework, report the assumptions made for the data analysis, and present the employed methodology to make all required steps transparent.

## Methods

### Participant and setting

It has long been known that alcohol is a toxic and harmful substance [16]. Its consumption contributes considerably to the global burden of disease [17]. Alongside long-term physical and mental health impairments [18–20], consuming alcohol may have acute effects such as mood changes [21].

The consumption of beer, wine, spirits and other alcoholic beverages is widespread in Europe [22]. In Germany, where the N-of-1 trial participant lives, the annual alcohol consumption per capita (aged > 15 years) equals 10.7 L of pure alcohol [23]. Given that no safe drinking levels can be established for young adults [24], public health campaigns, policies and other initiatives aim to reduce the overall levels of alcohol consumption in the population. Voluntary alcohol abstinence campaigns seek to encourage the general population to restrain from alcohol for a certain period of time [25]. Those who accept the challenge of temporarily abstaining from alcoholic drinks are given the opportunity to reflect on their drinking behavior and potentially reduce consumption afterwards [25]. In Europe, one-month abstinence campaigns were initiated in 2013 in the United Kingdom (Dry January®) [26, 27], in 2015 in the Netherlands (“IkPas”) [28], in 2016 in Hungary (“Száraz November”) [29], in 2017 in Belgium (“Tournée Minérale”) [30–32], and in 2020 in France (“Le défi de janvier”) [33]. In 2023, the Blaues Kreuz in Deutschland e. V. started a one-month abstinence campaign in Germany for the month of January, called “Trockener Januar” [34].

The participant had heard about the German voluntary one-month alcohol abstinence campaign from his peers and decided to voluntarily abstain from alcohol for the month of January 2024, in adherence with the campaign. The participant was a healthy male in his early 30 s, living in Berlin (Germany), and this was the first time he had adhered to an alcohol abstinence campaign. The participant obtained a score of 5/12 in the Alcohol Use Disorders Identification Test-Consumption screening instrument (AUDIT-C) [35], indicative of possible hazardous alcohol use; held a tertiary qualification, and he rated his social status using the MacArthur scale [36] as 6/10, indicating a relatively high socioeconomic status for an adult male in Germany [37].

In a conversation with one of the authors, the participant expressed his interest in measuring the effects of alcohol abstinence on his mood. The participant then designed, with the help of the author, a data collection system to collect all information deemed relevant. The decision to join the voluntary one-month alcohol abstinence campaign was an autonomous decision of the participant, which he made prior to deciding to set up a study to investigate the effect of alcohol abstinence on mood. However, the scheduled abstinence period can be considered as an “intervention” due to its well-established and external nature. The treatment schedule for the study (i.e., start and end dates of the treatment period) was externally defined by the organizers of the “Trockener Januar” campaign [34]. Therefore, this one-participant study fulfills our working definition of an N-of-1 trial. Moreover, the participant initiated the study and, with the technical assistance of an author, defined the precise research question, the design of the study, and the scale/timing of measured variables. Given the central role of the participant, this study represents an example of individual-centric N-of-1 trial.

### Study design

In this N-of-1 trial, the intervention “alcohol abstinence” and the comparator “no intervention” were compared. The intervention was defined as no consumption of beverages containing more than 0.5% of alcohol (the upper limit for beverages labeled as “alcohol free” in Germany). The data collection started on December 6, 2023 and ended on March 2, 2024. Between January 1, 2024 and January 31, 2024 the participant was in the intervention period, and abstained from alcohol. Outside the intervention period, the participant did not self-impose abstinence and could drink alcoholic beverages freely. We informally refer to the comparator as “drinking as usual”.

### Data collection

The participant decided to report information about key variables three times per 24-h period; specifically, in 8-h increments, at 00:00 (midnight), 08:00, and 16:00, every day. Data were collected using an electronic spreadsheet that the participant could access using his mobile phone. Data entered in the previous days were automatically hidden (colored as the background by an automatic function), to avoid influencing the participant’s reporting. A silent cellphone alarm reminded the participant to enter data when required, alerting him during the full study period at the three prespecified measurement times. At each measurement time, the participant recorded mood levels, number of alcohol units consumed, and presence of any social interactions. All measurements referred to the experience of the participant in the previous 8 h, that is, during the time period between the last measurement time and the current one. Mood levels (outcome variable) were recorded, in accordance with the preferences of the participant, on a 5-point Likert scale, ranging from −2 to 2, with higher values indicating a better mood. One alcohol unit was defined as one bottle/glass of beer (0.33 L), one glass of wine (0.1 L), or one shot of liquor (0.045 L). Social interaction was recorded as a binary variable (yes/no), indicating whether the participant met at least one other person in a social context, except for meeting his cohabitating partner at home. For the duration of the study, the participant could reach out to one of the authors to ask for technical assistance with the data collection system.

A time point was attributed to the intervention period if the 8 h preceding its measurement time fell into the scheduled month of alcohol abstinence. Additionally, the participant reported if the data entry happened before (i.e., more than 10 min before) or after (i.e., more than 10 min later) the pre-specified data collection time. Earlier or later reporting was inevitable if, for example, the participant was sleeping or could not use his phone precisely at the pre-specified time. In such instances where the reporting happened more than 10 min after the scheduled measurement time, the participant entered data based on his best recollection of the past. While many of his peers knew about the individual’s decision to adhere to the alcohol abstinence campaign, they were not informed during the study about the ongoing data collection. At the end of the study, information on whether the measurement time happened to fall on a pre-established non-working day for the participant (i.e., public holidays, vacation days or weekends) was also collected.

### Statistical analysis

In order to analyze the N-of-1 trial data, we relied on the explicit causal framework proposed by Robins et al. [38], and discussed specifically for N-of-1 trials in Piccininni et al. [14]. We conceived the existence of a variable, indicated with the letter U, that affects all outcome and time-varying covariate measurements [14]. The variable U can be informally interpreted as an “individual-specific variable”, summarizing the state of the individual at the beginning of the trial [14, 38]. In an N-of-1 trial, all observations are conditioned, by design, on the value of the variable U for the participant. We, therefore, aimed at estimating the average treatment effect of alcohol abstinence conditional on U, also called U-CATE [14]. The U-CATE is the individual-specific difference between the expected outcome under the treatment strategy “always intervene” and the expected outcome under the treatment strategy “never intervene” at a given time [14]. In our study, this corresponds to the difference in the expected mood levels between always abstaining from alcohol versus always drinking as usual.

**Fig. 1:**
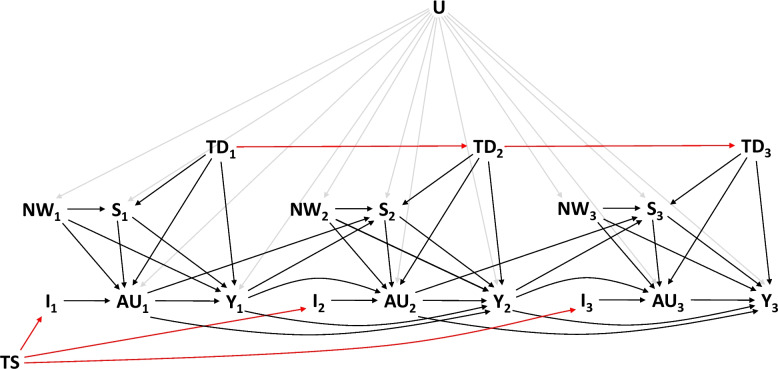
Directed acyclic graph representing the assumed data generating process for the first three measurement times. Subscripts indicate measurement times. The graph represents the assumed causal relationships between the treatment schedule (TS), the intervention status (abstinence or drinking as usual; I), the number of alcohol units reported (AU), the binary variable indicating whether it was a non-working day (NW), the binary variable indicating whether a social interaction occurred (S), the time of day (0:00, 8:00, 16:00; TD), the mood level (Y), and the individual-specific variable (U). Red edges indicate fully deterministic relationships, while edges from the individual-specific variable are colored in grey to avoid visual clutter

To estimate the U-CATE, we made several assumptions about the data generating process. Some of the assumptions about the relationships between the collected variables are represented in the causal Directed Acyclic Graph in Fig. 1.

First, we considered the treatment schedule, that is, the sequence of intervention and no intervention, as externally determined and fixed. Second, we assumed that having had social interactions at a given measurement time was only determined by the individual-specific variable, whether it was a non-working day, the time of the day, the mood level and the consumed alcohol units reported at the immediately preceding measurement time. We then assumed that the conditional distribution of the (counterfactual) social interaction variable was stationary [14]. Informally, the stationarity assumption states that if the individual were to be observed with the same values for the direct causes of social interaction at two different times, the probability of having a social interaction at these two times would be the same. Loosely speaking, this means that the conditional distribution of social interaction does not change over time. We then learned this conditional distribution by fitting a logistic regression model with social interaction (yes/no) as the dependent variable and all of its assumed direct causes as independent variables (except U, which is conditioned on by design).

Third, we assumed that, when the participant was not in the intervention period, the units of alcohol consumed reported at a given measurement time were only determined by the individual-specific variable, the presence of social interaction at that time, whether it was a non-working day, the time of the day, and the mood levels reported at the immediately preceding measurement time. We assumed stationarity also for the distribution of the variable alcohol units conditional on its direct causes, and we modeled this relationship using a zero-inflated Poisson regression model. This model choice reflects the idea that the participant, at each time, had a nonzero probability, dependent on the values of the covariates, of being in a setting in which he did not consume alcohol.

During the intervention the participant did not consume any alcohol; therefore, we assumed that under intervention the alcohol units are always zero.

Fourth, mood levels at a given time were assumed to be directly caused by the individual-specific variable, alcohol units consumed at that time, the presence of social interactions at that time, whether it was a non-working day, the time of the day, and the mood levels and alcohol units reported at the immediately previous measurement time. We assumed stationarity also for the distribution of mood conditional on its direct causes, and we modeled this relationship using an ordinal logistic regression.

Finally, we considered the data observed in the first time point as fixed, so that the direct causes of all measurements were available [14]. Consequently, the sequence of values for the variable “time of the day” was also fixed and did not require modeling. We also considered the sequence of values indicating whether the day was a non-working day to be fixed. Indeed, we can assume that the determination of weekends, national holidays, and choice of holidays was established before the start of the study. Therefore, we estimated the individual-specific causal effect, conditional on the sequence of working days and the first time point, for every time point from the second measurement time to the end of the study.

To estimate the individual-specific effect (U-CATE), we used a g-computation Monte Carlo algorithm [39]. For this, we used the three regression models and the deterministic relationships described above to sequentially generate data at each time point under a “always intervene” and “never intervene” treatment strategy. The mean of the outcome difference between the two scenarios for a specific time, across 800 repetitions, represents the point estimate for the individual-specific effect at that measurement time.

The 95% confidence intervals were obtained by parametric bootstrapping. Using the original fit of the regression models for social interaction, consumed alcohol units, and mood, we generated 500 bootstrapped datasets under the treatment assignment observed in the actual data. We then repeated the procedure to obtain the individual-specific effect point estimate for every time in each bootstrapped dataset. We used the standard deviation of the effect estimates at each time point across the bootstrapped datasets as an approximation of the estimator’s standard error. We subsequently used this standard error to obtain 95% confidence intervals by normal approximation.

All analyses were conducted in R v4.3.3 and RStudio v2023.12.1 + 402. Binary and ordinal logistic regression models were fit using the “lrm” function from the “rms” package, whereas zero-inflated Poisson regression models were fit using the “zeroinfl” function from the “pscl” package.

## Results

Overall, 264 time points were recorded, 171 (64.8%) under no intervention, and 93 (35.2%) during the intervention period. Information about all time-varying variables was complete (no missing values). Information was reported within 10 min of the scheduled time 92 (53.8%) times in the non-intervention period and 45 (48.4%) times in the intervention period. During the non-intervention period, the participant reported having consumed an average of 1.01 alcohol units per 8 h, while no alcohol units were reported during the intervention period, i.e. the participant perfectly adhered to the assigned intervention. The average mood levels were slightly higher during the intervention period (0.54 ± 0.87) compared to the non-intervention period (0.47 ± 0.96), but this observed difference does not necessarily represent a causal effect. For example, compared to the intervention period, during the non-intervention period a higher number of time points were recorded on non-working days (42.7% vs. 31.2%) and after social interactions (27.5% vs 15.1%, see Table 1).

**Table 1:** Descriptive statistics for the time-varying variables by intervention period

	**Non-intervention period (*N* = 171)**	**Intervention Period (*N* = 93)**
Non-working day, n (%)	73 (42.7%)	29 (31.2%)
Social interaction, n (%)	47 (27.5%)	14 (15.1%)
Alcohol units, mean (SD)	1.01 (2.35)	0.00 (0.00)
Mood, mean (SD)	0.47 (0.96)	0.54 (0.87)
Early reporting, n (%)	15 (8.8%)	9 (9.7%)
Late reporting, n (%)	64 (37.4%)	39 (41.9%)

Coefficients for the regressions modelling the conditional distributions of social interaction, alcohol consumption, and mood are reported in Table 2. We found that the probability of social interactions was higher on non-working days compared to working days (β = 1.49; 95%CI: 0.74, 2.23) and in the evening (Table 2). When in a drinking setting, the number of reported alcohol units was higher on non-working days compared to working days (β = 0.67; 95%CI: 0.25, 1.09) and after social interactions (β = 0.89; 95%CI: 0.21, 1.57), while it was lower in the morning (β = −1.21; 95%CI: −2.12, −0.30). The participant’s mood levels were higher during non-working days, in the evening, after social interactions, and increased with the mood levels reported in the previous time point (Table 2). No statistically significant effect of alcohol units on mood level was found for measurements at the same time point (Table 2). However, the number of alcohol units reported at the previous time point had a statistically significant negative effect on mood levels (β = −0.16; 95%CI:−0.29, −0.02; Table 2).

**Table 2:** Coefficients of the regression models used to model the data generating process. Rows list the independent variables in the regressions; columns correspond to the dependent variables. A logistic regression was used to model social interaction; a zero-inflated Poisson regression was used to model alcohol consumption (zero-inflation and count components are reported separately); and an ordinal logistic regression was used to model mood. The alcohol consumption model was only fit using data from the non-intervention period, as zero alcohol units were consumed in the intervention period. The horizontal dash indicates that the independent variable was not included in the model. 95% confidence intervals are presented in brackets and were obtained by normal approximation

	**Social interaction model**	**Alcohol model (zero-inflation)**	**Alcohol model (count)**	**Mood model**
Alcohol units - previous time	0.18 (−0.05, 0.42)	-	-	−0.16 (−0.29, −0.02)
Mood - previous time	−0.21 (−0.62, 0.19)	−0.01 (−0.91, 0.89)	0.01 (−0.17, 0.19)	0.71 (0.42, 1.00)
Non-working day	1.49 (0.74, 2.23)	−1.4 (−3.47, 0.67)	0.67 (0.25, 1.09)	1.22 (0.64, 1.80)
Time 00:00 (ref.)	-	-	-	-
Time 8:00	−3.57 (−5.10, −2.04)	13.06 (−257.71, 283.82)	−1.21 (−2.12, −0.30)	−1.43 (−2.16, −0.71)
Time 16:00	−1.42 (−2.15, −0.68)	25.36 (−389.43, 440.16)	−0.28 (−0.82, 0.26)	−1.15 (−1.79, −0.50)
Social interaction	-	−24.18 (−438.96, 390.61)	0.89 (0.21, 1.57)	1.31 (0.55, 2.08)
Alcohol units	-	-	-	0.03 (−0.12, 0.19)

The extreme regression coefficients and standard errors in the zero-inflation model indicate quasi-complete separation [40]. Indeed, in the non-intervention period, the participant never consumed alcohol between 00:00 and 16:00 without having experienced social interactions; and he always consumed some alcohol in the 8 h before midnight of a non-working day, when social interactions happened. Representing a likely structural deterministic pattern, the model predictions could still be useful in practice despite the large standard errors. We visually compared the observed time series of the modeled time-varying variables with 100 simulated time series generated using the regression models, and concluded that the regression models were a reasonable approximation of the data generating process.

**Fig. 2:**
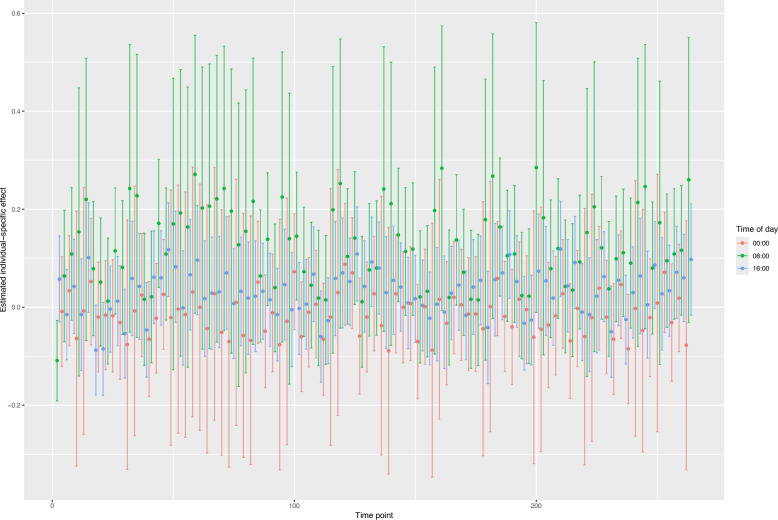
Estimated individual-specific effect of alcohol abstinence on mood levels (U-CATE) over time. At each time point, the estimate represents the estimated individual-specific difference between expected mood levels under the “always abstaining” strategy and the “always drinking as usual” strategy. Point estimates were obtained using a g-computation Monte Carlo algorithm, while 95% confidence intervals (vertical bars) were obtained by parametric bootstrapping

The estimated individual-specific effect on mood levels of “always abstaining” versus “drinking as usual” (U-CATE) is reported in Fig. 2 for each time point. At each time point, a positive U-CATE indicates higher expected mood under the “always abstaining” strategy compared to “always drinking as usual”. The average of the individual-specific effects estimates across the whole study period was 0.05 (95%CI: −0.06, 0.15). While this overall effect was not statistically significantly different from zero, it is interesting to notice that the point estimates have different signs depending on the time of the day. Indeed, the average of the individual-specific effects was −0.01 (95%CI: −0.16, 0.13) for measurements at midnight and 0.13 (95%CI: −0.04, 0.29) for measurements in the morning.

## Discussion

N-of-1 trials can be truly individual-centric studies in which the participant, through self-experimentation, answers causal questions that are relevant to them. This study design can “awaken patients’ inner scientists” and give patients more control over their health [41]. To illustrate how individual-centric N-of-1 trials can, if properly analyzed, be used to answer relevant causal questions, we presented a case study. Specifically, we described the design and analysis of an N-of-1 trial to assess the effect of alcohol abstinence on mood for a young male living in Germany, using data collected during his participation in a voluntary, month-long alcohol abstinence campaign.

### Case study in context

Experimental research on the effects of alcohol abstinence on health-related outcomes is rare. An RCT on the effect of preoperative alcohol abstinence on postoperative outcomes found that patients with alcohol use disorder who abstained for a month before surgery showed less postoperative morbidity compared to those who continued drinking alcohol [42]. Although the effects of alcohol abstinence can be analyzed using RCTs [43], this remains a challenging task [25]. Causal inference from observational studies has also proven challenging, as those who abstain from alcohol are generally not comparable to the general population. For example, compared to moderate drinkers, abstainers are on average older and in poorer health [44], and are more likely to have a history of alcohol-related hospitalization [45]. Some authors believe this strong unmeasured confounding, called abstainer bias, to be the explanation of the J-shaped relationship between alcohol consumption and mortality. The typical J-shaped relationship between alcohol and negative health outcomes has led researchers to consider a protective effect of low alcohol consumption plausible [46]. However, several meta-analyses [47–52] and an umbrella review of systematic reviews [53] have refuted this claim, pointing to abstainer biases as an alternative explanation.

Previous observational studies indicate that temporary abstinence during alcohol campaigns could have multiple positive health effects for participants. These include decreases in subsequent alcohol consumption [54–57], increases in general self-efficacy [58], drink refusal self-efficacy [54, 57, 59], self-reported physical and mental wellbeing [54, 58, 60], as well as improvements in physical biomarkers [61]. In contrast, one study suggests that increases in Dry January participation were not associated with lower levels of alcohol consumption on a population level between 2015 and 2018 [62]. Others even argued that abstinence challenges may do more harm than good [63]. Unintended consequences of voluntary temporary alcohol abstinence may be social exclusion or stigmatization of peers [64] or, for people with higher alcohol dependence severity, serious alcohol withdrawal symptoms [65]. It is therefore plausible that alcohol abstinence has different effects depending on the characteristics of the individual, justifying the implementation of an N-of-1 trial to quantify the individual-specific effect.

### Main findings

In our N-of-1 trial, the participant consumed more alcohol on social occasions, in the evenings, and during non-working days. We found evidence for an effect of number of alcohol units consumed at the previous measurement time (consumed more than 8 h before) on mood levels, but no evidence for an effect of the number of alcohol units consumed in the previous 8 h, adjusting for previous mood, time of day, whether it is a non-working day, and social interactions. Comparing “always abstaining from drinking” to “always drinking as usual”, we found no evidence of an average mood difference across the entire study period. This result could be explained by the participant’s relatively low habitual alcohol consumption. However, our results also seem to suggest that alcohol abstinence had modest positive effects on the mood in the mornings.

The effects estimated in our N-of-1 trial are individual-specific, and do not necessarily generalize beyond the participant. It seems, however, biologically plausible that alcohol abstinence improves mood in the mornings for certain individuals. While it is known that alcohol can regulate negative emotions during consumption [21], it also can have a particularly negative effect on mood the next day [66]. For example, an online survey among young adults who recently had a hangover reported that participants perceived their hangover symptoms, such as tiredness, sleepiness, or headache, as having a negative impact on their mood [67]. Naturalistic within-subjects studies also showed that young social drinkers experience more negative mood [68, 69] and lower emotional regulation [70] the day after alcohol consumption compared to days after no alcohol consumption. Consistently, a placebo-controlled randomized cross-over study indicated that young adults who were randomized to alcoholic beverages reported worse moods the next day [71].

### Strengths and limitations

In our work, we relied on a large dataset with high quality data. We had more than 260 measurements from a single individual, with measurements taken multiple times per day over a period of almost three months. Information was self-reported by the participant in a timely manner, and there were no missing values in any of the variables. This was possible only because of the participant’s strong motivation to answer the research question and because the study was tailored to the participant’s needs and lifestyle. N-of-1 trials are indeed a patient-centered and user-centered design that allows the participant to personalize the study design, choose the research question, and define an outcome that is relevant to them [5, 13].

In order to estimate the individual-specific effect of alcohol abstinence on mood, we relied on a explicit causal framework [14]. This allowed us to clearly define the estimand of interest and the assumptions made. We modeled the complex relationships of the assumed data generating process. Indeed, the relationship between mood and alcohol consumption is a complex one. Drinking not only affects mood, but mood also affects alcohol consumption [72]. Moreover, having social interactions and not working on a specific day are common causes of both alcohol consumption [73, 74] and mood levels [75, 76]. We estimated the effect of alcohol abstinence on mood by modeling the consumed alcohol units when the participant did not abstain, and separately, the mood levels conditional on the assumed direct causes (including the alcohol units consumed). This approach has similarities with the well-known front-door criterion [1, 2, 8, 77], and is justified by the fact that, in our causal model, we assumed consumed alcohol units to be the full mediator of the effect of alcohol abstinence on mood levels.

Some limitations need consideration when interpreting our results. Some of these limitations are inherent to the causal framework chosen for the analysis. First, our results hinge on the fact that the assumed causal model correctly represents the true data generating process. We assumed that, for the participant, only a few non-exogenous variables were causes of social interactions, alcohol consumption, and mood. It is possible that this assumption may not always hold. For example, we know that the participant was sick for three days during the intervention period, which may have had an effect on the participant’s mood. We additionally assumed that only other variables measured in the same time point or in the previous time point were direct causes of time-varying variables. We also relied on an assumption of stationarity of the conditional counterfactuals for all time-varying variables [14]. Informally, we assumed that the underlying data generating process of every time-varying variable was stable for the entire duration of the study, implying that the distribution of a time-varying variable would be the same over time for the same values of its direct causes. This assumption was important for our approach, as it allowed us to reconstruct, leveraging information from other observed time points, what would have happened to the participant at a fixed time under different circumstances than the ones under which he was actually observed. Stationarity is a strong assumption, often needed for proper causal inference in N-of-1 trials [14, 78] but is not always plausible. For example, we assumed that the behavior of the participant after the voluntary abstinence period did not fundamentally change, in the sense that the alcohol consumption distribution conditional on its direct causes remained the same as before the campaign. However, we emphasize that conditional stationarity does not imply marginal stationarity; therefore, for example, our model is compatible with a different probability of drinking alcohol at two time points (e.g., before and after the abstinence period) if the distribution of the direct causes is different at those times. Furthermore, we modelled the time-varying variables using parametric models and estimated the individual-specific effects and confidence intervals relying on Monte Carlo algorithms. Therefore, our estimates’ validity hinges on correct model specification and on the fact that the number of Monte Carlo algorithm runs was sufficiently high. We performed some visual checks for the goodness of fit of the parametric models, and chose a number of iterations that was high but reasonable in terms of computational costs.

Finally, we cannot rule out measurement error. It is especially possible that, since the data is self-reported, the amount of alcohol units consumed is not correctly inserted due to the effects of alcohol itself. Future N-of-1 trials could use breathalyzer to measure alcohol concentrations in the breath [79] and focus on more objectively measurable health outcomes, such as sleep quality measured using a wearable device [80].

## Conclusion

N-of-1 trials can be designed to be truly individual-centric studies, tailored to the needs and preferences of the participants. The basic idea of N-of-1 trials is to compare outcomes under intervention and no intervention for the same individual. Despite the simple idea, analyzing data from N-of-1 trials can be quite difficult when dealing with complex phenomena. A clear causal framework can help clarify the causal question of interest and make the assumptions made in the analysis phase explicit.

## Data Availability

The dataset analysed in the current study is not publicly available due to privacy concerns. The dataset is available from the corresponding author upon reasonable request, provided the participant has given their permission for data sharing.
